# Phytofabrication of Silver/Silver Chloride Nanoparticles Using Aqueous Leaf Extract of *Oedera genistifolia*: Characterization and Antibacterial Potential

**DOI:** 10.3390/molecules24234382

**Published:** 2019-11-30

**Authors:** Kunle Okaiyeto, Mike O. Ojemaye, Heinrich Hoppe, Leonard V. Mabinya, Anthony I. Okoh

**Affiliations:** 1 SAMRC Microbial Water Quality Monitoring Centre, University of Fort Hare, Alice 5700, South Africa; mojemaye@ufh.ac.za (M.O.O.); lmabinya@ufh.ac.za (L.V.M.); aokoh@ufh.ac.za (A.I.O.); 2 Applied and Environmental Microbiology Research Group (AEMREG), Department of Biochemistry and Microbiology, University of Fort Hare, Alice 5700, South Africa; 3 Department of Chemistry, University of Fort Hare, Alice 5700, South Africa; 4 Department of Biochemistry and Microbiology, Rhodes University, Grahams Town 6140, South Africa; H.hoppe@ru.ac.za

**Keywords:** *Oedera genistifolia*, green synthesis, Ag/AgCl NPs, structural characterization, antibacterial activity

## Abstract

In this present study, silver nanoparticles (Ag/AgCl NPs) were synthesized using an aqueous leaf extract of *Oedera genistifolia* as a reducing agent. The biosynthesized Ag/AgCl NPs was characterized by UV-visible spectrophotometry, transform infrared spectroscopy (FTIR), scanning electron microscopy (SEM), energy dispersive X-ray spectroscopy (EDX), transmission electron microscopy (TEM), X-ray diffraction (XRD), and thermogravimetric analysis (TGA). In addition, sequel to antibacterial assay, the cytotoxic effect of the phytofabricated Ag/AgCl NPs was assessed against the HeLa cell line (human cervix adenocarcinoma). The results of the characterization of the synthesized Ag/AgCl NPs indicate the successful synthesis using plant extract as a reducing agent, with UV-Vis spectra between 290–360 nm. TEM results showed that Ag/AgCl NPs was spherical in shape with an average size of 34.2 nm. EDX analysis revealed that the particles were predominantly composed of carbon, oxygen, chlorine, and silver, while FTIR identified major phytochemical compounds, which could be responsible for bio-reducing and capping potential. XRD analysis showed the crystallinity of Ag/AgCl NPs, with a face-centred cubic structure. The studied Ag/AgCl NPs had no cytotoxic effect on HeLa cells and exhibited antibacterial activity (minimum inhibitory concentration (MIC) 0.25–1 mg/mL; minimum bactericidal concentration (MBC) 2–16 mg/mL) against both the Gram-negative and Gram-positive bacteria investigated. Findings from this study suggest that this plant as a good candidate for producing new antibacterial drugs.

## 1. Introduction

Nanotechnology has recently become a subject of active research in materials chemistry. This field deals with the synthesis of particles with structural dimensions on the nanometre scale [1,2,3]. Nanoparticles of metal origin such as silver have been widely used in the synthesis of products such as anti-cancer, antioxidants, and antibacterial agents, as well as in cosmetics, due to their unique superior features [4,5]. The utilization of substrates such as bacteria, plant extracts, biodegradable polymers, and fungi for the synthesis of silver nanoparticles offers several advantages such as environmental friendliness, and compatibility with industrial and biomedical applications, because of the exclusion of harmful chemicals in their synthesis procedures [6,7]. In addition, these protocols involve simple reproducible steps that often result in stable, flexible materials, which do not require high pressure, energy, or temperature inputs, and can be effortlessly adapted for industrial scale production [8,9,10].

Although, in recent times, microorganisms have also been employed in the synthesis of nanoparticles, their synthesis rate has been reported to be slower as compared to plant-mediated synthesis [11,12]. More so, many researchers prefer plant-mediated nanoparticle synthesis compared to microbe-based synthesis, due mainly to its cost-effectiveness as well as having no requirement for time-consuming maintenance of microbial cultures [13]. The biological applications of silver nanoparticles have been effective in combating many dreadful diseases, especially those caused by multi-drug resistant pathogens [14]. In addition, silver nanoparticles have been reported to possess antibacterial, anti-fungal, as well as anti-cancer properties [15]. Previous studies have shown that they are not cytotoxic to humans, and are effective against fungi, viruses, and bacteria at low concentrations, with minimal or no side effects [16].

Recently, nano-encapsulated therapeutic agents have been utilized to selectively target anti-tumour agents, thus, resulting in higher drug concentrations at the tumour site [17]. However, the exact mechanism of silver nanoparticles derived from plant origin has not yet been extensively elucidated, and consequently, their mechanism of action is not yet fully understood. Studies carried out by some researchers have revealed that phytochemical compounds such as phenolic, flavonoids, carbohydrates, terpenoids, and proteins act as reducing agents for the synthesis of nanoparticles, and their stabilization [17,18,19,20,21].

The incidence of antimicrobial and antibiotic drug resistance have long been impediments to employing effective control measures against infections, thus, compromising human health. Due to the pressing need for novel antibiotics, there has been a growing interest in research on medicinal plants, because they are prospective sources of therapeutic value, and a remarkable number of novel drugs have originated from them over the years [22]. As a result, the utilization of plants in nanoparticle synthesis has gained popularity due to their advantages over the conventional methods. In view of plant diversity, there is an urgent need to explore plants for the rapid synthesis of nanoparticles in order to reduce production costs.

In an effort to contribute to the green route synthesis of silver nanoparticles, the present study reports on a rapid, simple, and single-step biosynthesis of silver nanoparticles using the aqueous leaf extract of *Oedera genistifolia* (Asteraceae). Furthermore, the biosynthesized silver nanoparticles (Ag/AgCl NPs) were characterized in detail and subsequently, their cytotoxic effect on HeLa cells, and their antibacterial activity against some Gram-positive and Gram-negative bacteria were investigated. To the best of our knowledge, this is the first study reporting on the antibacterial activity of silver nanoparticles synthesized from aqueous extracts of *O. genistifolia*.

## 2. Results and Discussion

### 2.1. Screening of Phytochemical Compounds

Plant mediated nanoparticle synthesis has become a subject of active research globally with different plant species being utilized for nanoparticles synthesis [23]. In this study, our findings revealed the presence of phenolics, alkaloids, flavonoids, carbohydrates, proteins, and saponins as shown in Table 1. The presence of these phytochemicals has been related to different biological activities, including anti-diabetic, anti-cancer, anti-hypertensive, and immunomodulatory activity [24]. Previous studies have revealed the utilization of leaf extracts from different plants such as *Allium sativum* [25], *Artemisia nilagirica* Ind. [26], *Tribulus terrestris* [27], *Mimusops elengi* [28], *Calliandra haematocephala* [29], and *Annona reticulate* [30] for silver nanoparticle synthesis. It has been previously described that phytochemicals present in the extracts are responsible for the reduction of metal ions, and efficient stabilization of nanoparticles, with the reduction of Ag^+^ to Ag^o^ ascribed to the presence of these phytochemicals in the aqueous leaf extract of *O. genistifolia* [22]. Most reports documenting nanoparticle synthesis have highlighted discrepancies in size, synthesis parameters, shape, and stability [31].

### 2.2. UV-Vis Spectroscopy Analysis

**Figure 1:**
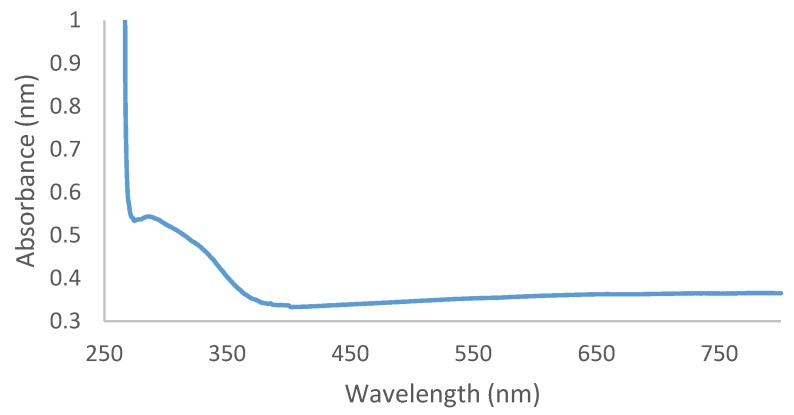
UV-Vis spectrometry analysis of silver nanoparticles (Ag/AgCl NPs) from aqueous leaf extract of *O. genistifolia*.

Ag/AgCl NPs synthesis via biological means using aqueous leaf extract of *O. genistifolia* was carried out in this study. Due to its high conductivity and catalytic properties, as well as its chemical stability, silver nitrate was used as a suitable precursor [32]. The gradual addition of the aqueous leaf extract of *O. genistifolia* (light brown colour) to silver nitrate solution resulted into the instant formation of a dark brown coloration, which could be ascribed to the completion of the reaction. For biological synthesis of nanoparticles to have added advantage over chemical methods, the time for synthesis is also a critical factor to be considered. Subsequently, UV–Vis spectroscopy was used to confirm the formation of the Ag/AgCl NPs, indicated by the appearance of the dark-brown colloidal solution, as a result of excitation of surface plasmon resonance (SPR) between 290–360 nm (Figure 1). Our findings corroborate with the absorbance value of biosynthesized silver nanoparticles reported from previous studies [33], and contrary to our present study, some biogenic AgNPs synthesized from other plant extracts had SPR between 400 and 500 nm [34,35,36]. The type of biogenic nanoparticles formed depends on the phytochemical compounds in the plant extract. The observed results show that the synthesis of Ag/AgCl NPs through the green approach in this study proved to be efficient with respect to reaction time, as well as having no requirement for the use of toxic chemical reducing or stabilizing agents. Therefore, making it an economical, sustainable, reliable, and an alternative process to chemical or physical methods for the synthesis of silver nanoparticles [12].

### 2.3. FTIR Analysis

**Figure 2:**
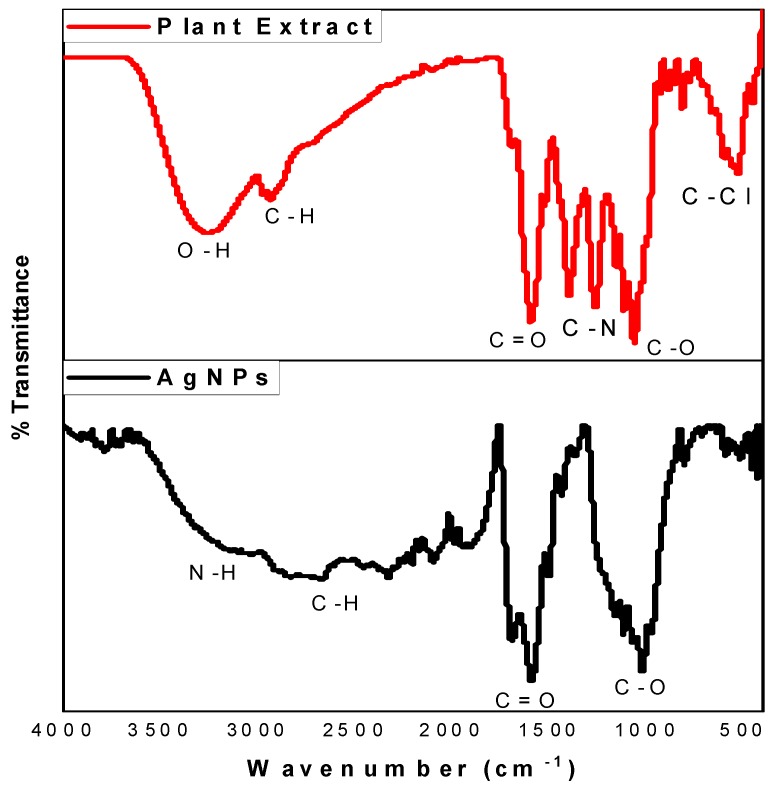
Transform infrared spectroscopy (FTIR) spectra of the aqueous extract of *O. genistifolia* leaf and Ag/AgCl NPs.

Transform infrared spectroscopy (FTIR) is a non-destructive, appropriate, indispensable, and simple method for the determination of the role of plant extracts in the reduction of silver ions to silver nanoparticles [37]. In this present study, the FTIR spectra of *O. genistifolia* extract and Ag/AgCl NPs were analysed, and a comparison of their spectra patterns (Figure 2). Vibrational frequencies at 1025, 1590, 2933 cm^−1^, etc., observed in the spectra of Ag/AgCl NPs and plant extract could be ascribed to the phytochemical compounds in the plant extracts which were not only responsible for the biosynthesis of Ag/AgCl NPs, but also acted as capping and stabilizing agents. This indicated that some of the residual moieties of the phytochemical compounds in the plant extract encapsulated the Ag/AgCl NPs, acting as capping and stabilizing agents [38]. Furthermore, a broad peak at 3454 cm^−1^ could be attributed to N-H stretching vibration of group NH_2_ and OH, in both *O. genistifolia* leaf extract and the Ag/AgCl NPs. The band at 1657 cm^−1^ from the plant extract corresponds to amide C=O stretching, which was also observed on the Ag/AgCl NPs at 1697 cm^−1^. Similarly, a peak at 1596 cm^−1^ could be assigned to the C=O group present in amide in the phytochemicals of the plant extract, which was also found on the Ag/AgCl NPs, with a slight shift in the spectra band at 1590 cm^−1^. The observed peaks at 1117 cm^−1^ (plant extract) and 1118 cm^−1^ (synthesized nanoparticles) denote -C-O-C- linkages, or -C-O- bonds. The detected peaks are mainly attributed to flavonoids and terpenoids present in the plant extract.

### 2.4. TEM, SEM and EDX Analyses

**Figure 3:**
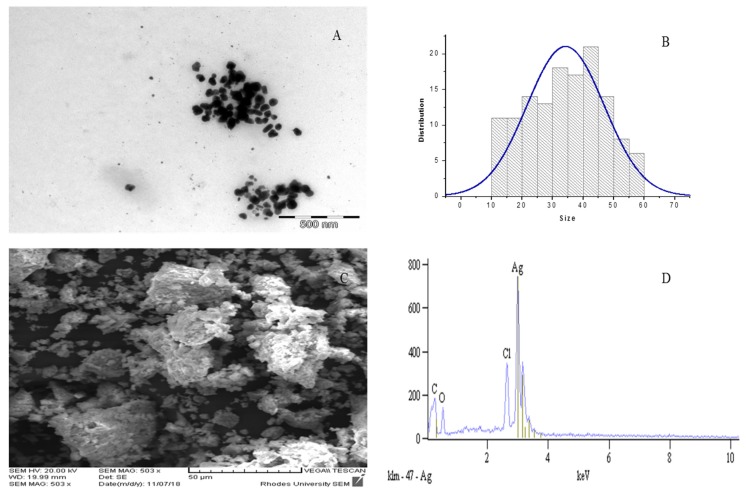
TEM image (**A**), Size distribution (**B**), SEM image (**C**) and energy dispersive X-ray spectroscopy (EDX) (**D**) analyses of biosynthesized Ag/AgCl NPs mediated by aqueous leaf extract of *O. genistifolia*.

TEM appears to be the most appropriate technique to determine the average size of nanoparticles; nevertheless, other techniques also contribute to understanding the salient features of nanoparticles [31]. The TEM analysis was used to describe the particles based on their shape, size, and particle dispersal. Previous studies have shown that the activity of green Ag/AgCl NPs is size-dependent, with smaller particles showing higher activity as compared to the larger ones [31]. Size distribution studies of the Ag/AgCl NPs synthesized in the present study indicated that the particles were of narrow size distribution, and fall in the acceptable limit range; thus, implying that the silver nanoparticles produced were relatively small. The results depicted in Figure 3A show the Ag/AgCl NPs as poly-disperse, predominantly composed of a well-defined spherical shape with a size range of 10–60 nm, with an average size of 34.2 nm without any agglomeration, and with some irregular rod-like shapes (Figure 3B). The silver nanoparticles synthesized from *Mentha pulegium* and *Prosopis cineraria* were reported to be in the range of 5–50 nm and 20–44 nm, respectively [39,40], which is similar to our result in this study. On the contrary, bigger sizes of silver nanoparticles of 90.87 nm and 74–94 nm have been documented from *Gelsemium sempervirens* and *Rosa damascene*, respectively [41,42]. Figure 3B depicts the size distribution image of Ag/AgCl NPs and the size distribution was observed to range from 10 to 60 nm. The calculated average particle size distribution of Ag/AgCl NPs was 34.2 nm. SEM was used to study the surface morphology of AgNPs in this present study. The Ag/AgCl NPs were observed to be uniformly distributed on the surface as illustrated in Figure 3C. The SEM images showed the shape of Ag/AgCl NPs as being non-consistent, with both spherical-shaped and triangular nanoparticles. These observations confirmed the results from the TEM analysis (Figure 3A) that revealed the spherical and irregular shapes of the Ag/AgCl NPs, which corroborate the previously reported studies [43,44]. The determination of the elemental compositions of the Ag/AgCl NPs was carried out by energy dispersive X-ray spectroscopy (EDX) analysis, to illustrate the surface morphological features of the Ag/AgCl NPs (Figure 3D). The presence of chloride ions in the *O. genistifolia* leaf extract was further confirmed by EDX and XRD analyses. The result confirmed the successful formation of Ag/AgCl NPs and revealed its predominant elements to include carbon (8.46%), oxygen (9.57%), chlorine (9.52%), and silver (72.44%). Apart from the intense peak of Ag observed at 3.0 keV, an intense peak of Cl was also noted at 2.7 keV. These intense peaks of Ag and Cl confirmed the formation of Ag/AgCl NPs, and our findings corroborate the reports of Ag/AgCl NPs synthesized from *Benincasa hispida* and *Momordica charantia* [45,46].

### 2.5. XRD Analysis

**Figure 4:**
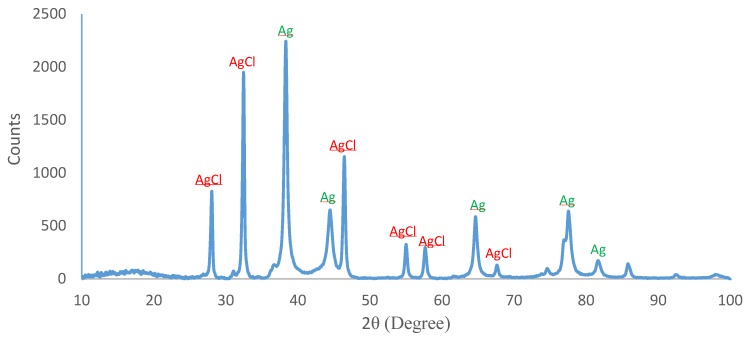
XRD results of the biosynthesized Ag/AgCl NPs from aqueous leaf extract of *O. genistifolia*.

XRD is generally used to elucidate the crystalline nature of Ag/AgCl NPs. It quantifies the firmness of diverse chemical compounds, thus indicating an idea of approximate different chemical groups and particle sizes. In this analysis, a monochromatic beam of X-rays was focused towards crystal samples, which subsequently produced different patterns that were analysed using Bragg’s equation in order to reveal the unique features of the crystallinity of the studied Ag/AgCl NPs [31]. In this present study, the crystalline nature of dry powder of the Ag/AgCl NPs was analysed by XRD. The XRD patterns (Figure 4) revealed five distinct diffraction peaks at 2θ degrees of 38.37° (111), 44.54° (200), 64.75° (220), 77.89° (311) and 81.87° (222), that evidently indicated the formation of the face-centred cubic (*fcc*) crystalline structure of the AgNPs, mediated by the aqueous leaf extract of *O. genistifolia*. The high intensity on this set of lattice planes displayed typical peaks of face-centred cubic (*fcc*) structure of AgNPs, which corresponded to the database of the Joint Committee on Powder Diffraction Standards (JCPDS), file No. 04-0783. Likewise, the Bragg reflections at 28.0°, 32.4°, 46.4°, 54.9°, 57.7°, and 64.7° could be indexed to the (111), (200), (220), (311), (222) and (400) planes, respectively (JCPDS, file: 31-1238) which indicate the *fcc*-structure of AgCl [47]. Similarly, our findings agree with the reports documented by other researchers [45,46,48,49,50]. As emphasized by the report of Davi et al. [46], at the initial stage of the reaction, AgCl formation at room temperature could be due to the reaction between Ag^+^ from AgNO_3_, and Cl^−^ from the phytochemical compounds in the aqueous leaf extract of *O. genistifolia*. Upon AgCl formation, Ag^+^ ions were reduced to metallic Ag by phytochemical compounds, which act as reducing agents originating from the plant extract. Subsequently, Ag^+^ formed an intermediate complex with OH groups of phenolic compounds from the extract, which later underwent oxidation and reduced Ag^+^ to Ag^o^ NPs.

### 2.6. TGA Analysis

**Figure 5:**
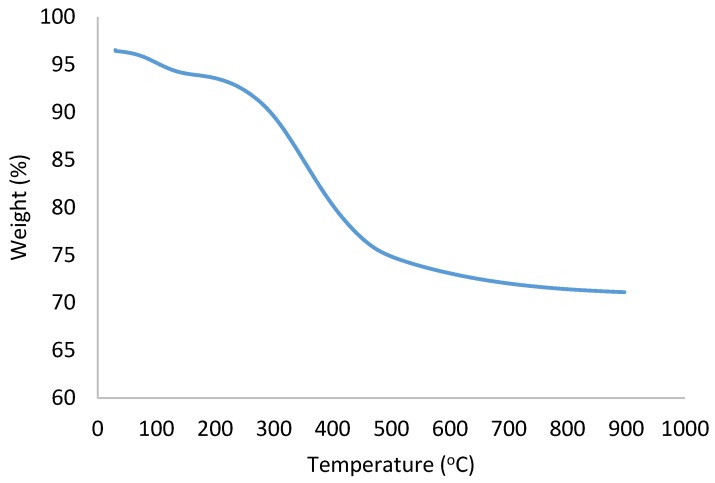
TGA analysis of the biosynthesized Ag/AgCl NPs from aqueous leaf extract of *O. genistifolia*.

The biosynthesized Ag/AgCl NPs retained more than 70% of their initial weight after heating up to 900 °C (Figure 5). The initial weight loss between 30–200 °C could be due to the loss of moisture content from the Ag/AgCl NPs, and further decrease in weight loss was observed. The Ag/AgCl NPs retained about 70% of its weight at 900 °C, which indicates its robustness nature. This indicates the stability of the synthesized Ag/AgCl NPs, and in addition, this salient feature makes it a good candidate for pharmaceutical applications and other industrial processes where high temperatures are involved.

### 2.7. Cytotoxicity Assay Against HeLa Cells

**Figure 6:**
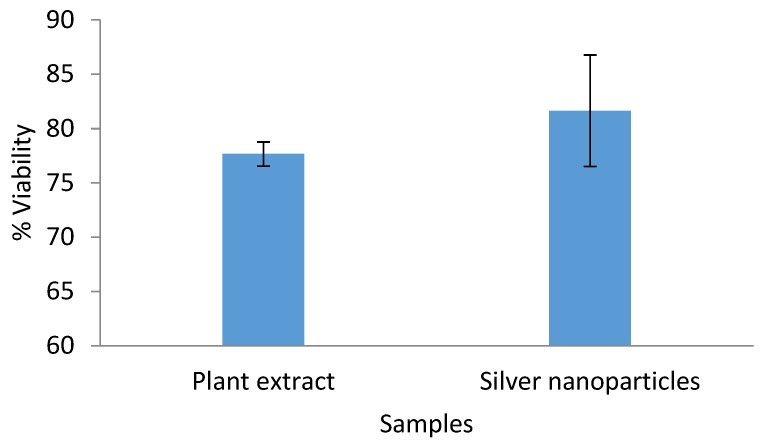
Percentage viability of HeLa cells incubated with 50 µg/mL aqueous leaf extract of *O. genistifolia* and Ag/AgCl NPs for 48 h. Error bars indicate standard deviation of triplicate wells.

Figure 6 depicts the cytotoxic effect of both the aqueous extract and the biosynthesized Ag/AgCl NPs, as assessed against HeLa cells. Both had no cytotoxic effect on the HeLa cells, since the cells viability was more than 75% in both samples (Figure 6), an indication of its safety for mammalian cells. This finding supports the safe utilization of the synthesized Ag/AgCl NPs as a lead substrate for drug design and development. However, some researchers in previous studies [17,51,52] have reported silver nanoparticles cytotoxic effects when synthesized from different plants.

### 2.8. Antibacterial Activity

Owing to the smaller particle size and recent medical advances, the use of silver nanoparticles against deadly diseases has gained significant interest as a means to overcome the limitations of orthodox treatments. The distinctive properties of silver nanoparticles coupled with the high surface area to volume ratio have rendered them potential candidates for biological activities, such as antimicrobial, anti-inflammatory, and anti-cancer activity [31]. In this study, the antibacterial potential of Ag/AgCl NPs mediated by an aqueous leaf extract of *O. genistifolia* was investigated as possible antibacterial agents against the following reference bacterial strains; Gram-positive *Listeria ivanovic* (ATCC 19119), *Streptococcus uberis* (ATCC 700407), *Staphylococcus aureus* (ATCC 29213), and *Mycobacterium smergatis* (ATCC 19420), and Gram-negative *Enterobacter cloacae* (ATCC 13047) and *Vibrio* sp. (polymerase chain reaction confirmed isolate).

The minimum inhibitory concentration (MIC) of Ag/AgCl NPs for the tested bacterial strains was 1 mg/mL for *L. ivanovic* (ATCC 19119), 0.5 mg/mL for *E. cloacae* (ATCC 13047), *S. uberis* (ATCC 700407), *S. aureus* (ATCC 29213), and 0.25 mg/mL for *M. smergatis* (ATCC 19420) and *Vibrio* sp., as shown in Table 2. The Ag/AgCl NPs had varying minimum bactericidal concentration (MBC) values for all the bacterial strains tested. Furthermore, the MBC of Ag/AgCl NPs for *E. cloacae* (ATCC 13047), *S. uberis* ATCC (700407), *S. aureus* (ATCC 29213) and *Vibrio* sp. were 16 mg/mL; whereas, they showed strong bactericidal effect at 8 mg/mL and 2 mg/mL for *L. ivanovic* (ATCC 19119) and *M. smergatis* (ATCC 19420), respectively (Table 1). On the other hand, the positive control, ciprofloxacin had a MIC of 0.25 mg/mL for all the tested bacterial strains, and a MBC of 1 mg/mL.

The antibacterial activity of the studied biosynthesized Ag/AgCl NPs was lower compared to the antibacterial activity demonstrated by the positive control, and this could be attributed to the fact that the crude extract of the studied plant was used in the synthesis of Ag/AgCl NPs and not the pure compound. However, the results from this assay showed that the studied Ag/AgCl NPs showed a promising antibacterial effect, and hence, it is highly desirable for future studies to isolate pure compounds from the aqueous extract and carry out further biological assay on them. Nevertheless, findings from previous studies have shown that the shape of nanoparticles influences the efficacy of their biological activity [53]; that is, spherically shaped Ag/AgCl NPs display superior antimicrobial activity compared to rod-like shaped nanoparticles. Therefore, it is noteworthy to observe that the results from the present study corroborate those reported by Moodley et al. [53] and Raja et al. [29], where spherically shaped Ag/AgCl NPs were documented with few rod-like shaped particles.

The mechanism of antibacterial activity of the studied Ag/AgCl NPs on the bacterial strains was not clearly understood. However, previous studies have reported that the bactericidal mechanism could be due to the action of Ag/AgCl NPs on the structural integrity of the cell membrane that often resulted in membrane permeability, and consequently led to cell death [54,55]. The results obtained from this study also indicated that aqueous leaf extract of *O. genistifolia* could be used for the natural and eco-friendly synthesis of silver nanoparticles, which could be used as a potential candidate species with antibacterial activity, and the mechanism of action would be highly relevant for future studies.

## 3. Materials and Methods

### 3.1. Plant Collection and Extraction

Fresh *O. genistifolia* leaves were collected around the vicinity (coordinates: 32.7885° S, 26.8461° E) of University of Fort Hare, Alice campus, South Africa, in the month of September 2018. The leaves were washed with running tap water to remove debris and other contaminating organic contents, were subsequently rinsed with distilled water, and air-dried at room temperature for 14 days. The dried plant sample was pulverized with an electric grinder, and 100 g of the powder was weighed into 500 mL of distilled water contained in a 1-litre bottle and heated in water-bath at 60 °C for 1 h. Thereafter, the mixture was filtered through Whatman no. 1 filter paper and the extract was stored at 4 °C for further use [12].

### 3.2. Screening for Phytochemical Constituents

The analysis of the phytochemical constituents in the aqueous leaf extract of *Oedera genistifolia* was carried out as described by Yadav and Agarwala [56] and Karthigaiselvi et al. [57], with slight modifications. The phytochemical compounds screened for included phenolics, tannins, flavonoids, alkaloids, saponins, terpenoids, glycosides, steroids, proteins, carbohydrates, and anthraquinone.

#### 3.2.1. Test for Phenols

We prepared fifty milligrams of Ag/AgCl NPs and added 5 mL of alcohol (50 mg/mL), which was treated separately with a few drops of neutral FeCl_3_ (NH_4_OH + FeCl_3_) solution. The sudden change in colour to blue-green or black coloration indicates the presence of phenols.

#### 3.2.2. Test for Alkaloids

The solution of Ag/AgCl NPs (50 mg/mL) was added to Mayer’s reagent (0.68 g of HgCl_2_ in 30 mL of distilled water, 2.5 g of KI in 5 mL of distilled water) solution. Formation of whitish yellow (or) cream coloured precipitate indicates the absence of alkaloids.

#### 3.2.3. Test for Tannins

Ag/AgCl NPs (50 mg/mL) was prepared, 1 mL was transferred in test tube, and 2 drops of 5% FeCl_3_ solution was added thereafter. Dirty green precipitate indicated the presence of tannin.

#### 3.2.4. Test for Flavonoids

Ag/AgCl NPs at a volume of 1 mL (50 mg/mL) was transferred into a test tube, and 3 mL of Pb(C_2_H_3_O_2_)_2_ solution was added. A bulky white Lead precipitate indicated the test was positive.

#### 3.2.5. Test for Saponins

Ag/AgCl NPs (100 mg) was mixed with 5 mL of distilled water in a test tube, and it was shaken vigorously. The formation of stable foam indicated the presence of saponins.

#### 3.2.6. Test for Glycosides

A volume of 2 mL glacial CH_3_COOH containing one drop of FeCl_3_ solution was added to 5 mL of Ag/AgCl NPs (50 mg/mL). A 1 mL volume of concentrated H_2_SO_4_ was poured along the sides of the tube. Formation of a brown ring at the interface indicated the presence of glycosides.

#### 3.2.7. Test for Terpenoids

Ag/AgCl NPs (50 mg) was added with chloroform and H_2_SO_4_. Red violet colour indicated the presence of terpenoids.

#### 3.2.8. Test for Protein

Ninhydrin solution (two drops of 10 mg of Ninhydrin in 200 mL of acetone) were added to 2 mL of Ag/AgCl NPs (50 mg/mL). A characteristic purple colour indicated the presence of amino acid.

#### 3.2.9. Test for Carbohydrates

Ag/AgCl NPs (50 mg) was mixed with 2 mL of iodine solution. A dark blue or purple coloration indicated the presence of the carbohydrate.

#### 3.2.10. Test for Anthraquinone

Ag/AgCl NPs (50 mg) was boiled with 10 mL of H_2_SO_4_ and filtered while hot. The filtrate was shaken with 5 mL of chloroform. The chloroform layer was transferred into another test tube. The resulting solution was observed for colour changes to violet indicating the presence of anthraquinine.

#### 3.2.11. Test for Steroids

Ag/AgCl NPs (50 mg) was taken in a test tube and dissolved with chloroform (10 mL), then had added an equal volume of concentrated H_2_SO_4_ to the test tube by sides. The upper layer in the test tube turned into a red and sulphuric acid layer showing yellow with green fluorescence, showing the presence of steroids.

### 3.3. Biosynthesis of Ag/AgCl NPs

The aqueous extract of *O. genistifolia* leaves was used to synthesize the green silver nanoparticles. Silver nitrate (A.R.) (99.2%, MW = 169.87) used for the synthesis was purchased from Merck, South Africa. A solution of silver nitrate (450 mL) (AgNO_3_) at 0.1 mM was prepared in 1 L of distilled water and 50 mL of the plant extract was added drop-wise into the AgNO_3_ solution; the mixture was stirred continuously for 1 h at ambient temperature and in a dark chamber to prevent photo-reduction of silver nitrate. The bio-reduction of Ag^+^ to Ag^o^ was confirmed by the physical colour change from colourless solution (silver nitrate solution) to a dark brown (Ag/AgCl NPs). The mixture was centrifuged at 15,000 rpm, 15 °C for 20 min to separate the pellets from the suspension, and 2 mL was withdrawn from the supernatant for UV-Visible spectroscopy analysis. Subsequently, the obtained precipitate was washed three times with distilled water in order to eliminate any other impurity that co-precipitated along with the Ag/AgCl NPs and later oven-dried at 80 °C overnight.

### 3.4. Characterization of Ag/AgCl NPs

The reduction of silver ions to Ag/AgCl NPs was monitored by using a UV-Vis spectrophotometer (Perkin-Elmer Lambda 25, Boston, MA, USA) from 250 to 800 nm. Subsequently, Fourier transform infrared radiation (Perkin-Elmer Universal ATR 100) was employed to identify the characteristic vibrational frequency of Ag/AgCl NPs [14]. Particle size and shape of Ag/AgCl NPs were determined using Transmission electron microscope (JEOL 1210, Austin, TX, USA), operated at an accelerated voltage of 100 kV. The surface morphology of nanoparticles was studied with SEM (JEOL JSM-6390 LVSEM) and the elemental compositions of Ag/AgCl NPs was carried out with Noran Six 200 Energy Dispersive X-ray (JEOL Ltd., Tokyo, Japan) [14]. XRD analysis was carried out by using X-ray Diffractometer to determine the crystalline nature of the biosynthesized Ag/AgCl NPs.

### 3.5. Cytotoxicity Assay—Single Concentration Screening

The cytotoxic effect of Ag/AgCl NPs was evaluated against HeLa cells (Cellonex, South Africa) as described by Larayetan et al. [33]. The sample was incubated at 37 °C for 48 h at a fixed concentration of 50 µg/mL in 96-well plates containing HeLa cells. Emetine was used as positive control drug and the cells that survived Ag/AgCl NPs and extract exposure were quantified by incubation with resazurin and measuring its conversion to resorufin by fluorescence (Exc_560_/Em_590_). Percentage viability of treated cells was calculated relative to fluorescence readings obtained with untreated control cells.

### 3.6. Antibacterial Activity Assay

Antibacterial activity of Ag/AgCl NPs was evaluated by micro-dilution procedure as described by Larayetan et al. [33] in order to determine the minimum inhibitory concentration (MIC). Mueller Hinton broth (MBH) (250 µL) was transferred into Eppendorf tubes for serial dilution. A stock solution (64 mg/mL) of Ag/AgCl NPs was prepared in dimethyl sulfoxide (DMSO) and subsequently, different concentrations ranging from 0.125–32 mg/mL were prepared by two-fold serial dilutions in MBH. Subsequently, 20 μL of fermented broth of each tested bacteria (0.5 McFarland 1 × 10^8^ cfu/mL) was added into the mixture and vortexed followed by incubating at 37 °C for 24 h. The positive and negative controls used were ciprofloxacin and 5% DMSO, respectively. Subsequently, the minimum bactericidal concentration (MBC) was determined by plating out those broths without visible growth on fresh Mueller Hinton agar, and further incubating the plates at 37 °C for 24 h.

## 4. Conclusions

The results from this study reveal that the aqueous leaf extract of *O. genistifolia* aided the synthesis of Ag/AgCl NPs in a one-step process that completely complied with the rules of green synthesis. The biosynthesis of Ag/AgCl NPs was confirmed by UV-Vis spectroscopy analysis and the XRD pattern of the produced nanoparticles revealed their crystalline nature, and the spherical shape of the typical nanoparticles with permissible sizes was revealed by TEM analysis. The studied Ag/AgCl NPs demonstrated good antibacterial potential against both Gram positive and Gram-negative bacteria with no toxic effect on HeLa cells, thus, revealing its safety for human use. The process of synthesis is environmentally compatible, and the synthesized Ag/AgCl NPs could be a promising candidate for development of new antibacterial drugs. Additionally, the findings from this study could serve as a basis for future studies on the mechanism of bactericidal effect of the synthesized Ag/AgCl NPs on the selected pathogens.

## Figures and Tables

**Table 1:** Qualitative screening of phytochemical constituents in the aqueous leaf extract of *Oedera genistifolia*.

Phytochemicals	Degree
Phenolics	+++
Alkaloids	+
Tannins	+++
Flavonoids	+++
Saponin	+++
Glycosides	−
Terpenoids	+
Steroids	+
Proteins	+
Carbohydrates	+
Anthraquinone	−

+++, Highly present; +, slightly present; −, not present.

**Table 2:** MIC and MBC of the Ag/AgCl NPs and ciprofloxacin.

Minimum Inhibitory Concentration (MIC)																		
Bacterial Strains	32 mg/mL		16 mg/mL		8 mg/mL		4 mg/mL		2 mg/mL		1 mg/mL		0.5 mg/mL		0.25 mg/mL		0.125 mg/mL	
	a	b	a	b	a	b	a	b	a	b	a	b	a	b	a	b	a	b
*Enterobacter cloacae (ATCC 13047)*	−	−	−	−	−	−	−	−	−	−	−	−	−	−	+	−	+	+
*Listeria ivanovic (ATCC 19119)*	−	−	−	−	−	−	−	−	−	−	−	−	+	−	+	−	+	+
*Streptococcus uberis* (ATCC 700407)	−	−	−	−	−	−	−	−	−	−	−	−	−	−	+	−	+	+
*Staphylococcus aureus* (ATCC 29213)	−	−	−	−	−	−	−	−	−	−	−	−	−	−	+	−	+	+
*Mycobacterium smergatis* (ATCC 19420)	−	−	−	−	−	−	−	−	−	−	−	−	−	−	−	−	+	+
*Vibrio* spp. (PCR confirmed isolate)	−	−	−	−	−	−	−	−	−	−	−	−	−	−	−	−	+	+
**Minimum Bactericidal Concentration (MBC)**																		
*Enterobacter cloacae (ATCC 13047)*	−	−	−	−	+	−	−	−	+	−	+	−	+	−	+	−	+	+
*Listeria ivanovic (ATCC 19119)*	−	−	−	−	−	−	+	−	+	−	+	−	+	+	+	+	+	+
*Streptococcus uberis* (ATCC 700407)	−	−	−	−	+	−	+	−	+	−	+	−	+	+	+	+	+	+
*Staphylococcus aureus* (ATCC 29213)	−	−	−	−	+	−	+	−	+	−	+	−	+	+	+	+	+	+
*Mycobacterium smergatis* (ATCC 19420)	−	−	−	−	−	−	−	−	−	−	+	−	+	+	+	+	+	+
*Vibrio* spp. (PCR confirmed isolate)	−	−	−	−	+	−	+	−	+	−	+	−	+	+	+	+	+	+

Note: a, Ag/AgCl NPs; b, ciprofloxacin; +, indicates growth; −, indicates no visible growth.
